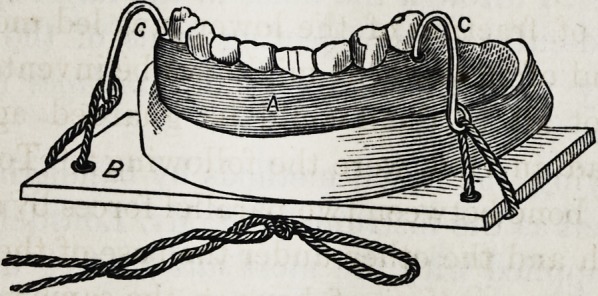# Treatment of Fracture of the Inferior Maxillary Bone by an Improved Apparatus

**Published:** 1869-12

**Authors:** Wm. G. Bullock

**Affiliations:** Professor of Surgery in the Savannah Medical College.


### ARTICLE VIII.

### *Treatment of Fracture of the Inferior Maxillary Bone by an Improved Apparatus.*

By WM. G. BULLOCK, M.D.,
Professor of Surgery in the Savannah Medical College.

(With a wood-cut).

Fractures of the lower jaw-bone and the difficulty of managing them have engaged my particular attention, from the fact of a number of such fractures having been under my observation in private practice, as well as in the practice of the hospital in this city, of which I have been one of the attending surgeons now over twenty-five years.

It has occurred to me to see fractures at several different points of this bone, but most frequently at or near the symphysis, or the middle of the chin, which is accounted for by its more exposed position. One was a case of double fracture, where the line of fracture ran just anterior to the insertions of the masseter muscle on either side, leaving the central portion detached and lowered by the depressor muscles of the chin. It seemed impossible to remedy the displacement in this case by the ordinary means taught in the schools, and described in surgical works for the treatment of this fracture. So also did the ordinary means fail in another case of double fracture through the symphysis and of the body near the angle. In neither of these cases was Gibson's, Barton's, or the four-tailed bandage, though faithfully tried, successful in keeping the fragments in satisfactory apposition, even with the assistance of a paste-board splint and compress, cork wedges grooved to fit the teeth, ligatures of thread or silver wire twisted around the teeth, or, lastly, the plan of Professors Mutter and Smith, of a leaden clamp or grooved plate fitted on the teeth.

I searched the many text-books and works on surgery in vain for a suitable apparatus to remedy the effects of the usual appliances in these cases Surgical writers have not, I think, given the subject proper attention. They tell the student of the usual seat, etiology, and indications for treatment of fracture of this bone, what muscles act in causing displacement, and how they are to be counteracted, then the subject is dismissed by the assertion that there is no difficulty in managing any of the varieties of this fracture if the student will only apply a compress, mould a piece of pasteboard, or of gutta percha to the jaw, and bandage the part. In fact, any means may be resorted to capable of carrying out the indications of restoring the line of the jaw, drawing it up to the superior maxilla, acting as a splint, to prevent displacement upwards, downwards, or laterally.

Mr. Fergusson, after alluding to various pieces of mechanism that have been devised for treating this kind of fracture, and simply mentioning the ingenious invention of Mr. Lonsdale, remarks, "For my own part I should commonly prefer the paste-board splint, cork, and bandage above recom mended, which is the mode usually employed." Now, I contend, that for the rapidity of cure, and for the comfort of a patient suffering from a fracture of the inferior maxilla, these usual plans are totally inadequate where our object should be to cure our patient "*tute et jucunde.*" I have often been consulted as to how the difficulties accompanying the treatment of this fracture could be best overcome and obviated.

Mr. Lonsdale says :—

"I would make the following objections to the usual treatment employed in fracture of the inferior maxillary bone. 1. In fractures by the molar teeth, the one portion of bone is often much pulled upon by the action of the pterygoid muscles ; that great displacement is produced inwards, and which the pieces of cork cannot overcome. 2. As the position of the fractured portions of bone depends upon the pressure that is made to keep the lower jaw fixed against the upper, the least loosening of the bandage or extra action of the muscles will be liable to disengage the pieces of cork, and to destroy the apposition of the ends of bone. 3. The necessity for keeping the lower jaw so firmly pressed against the upper is very uncomfortable and irksome to the patient by preventing him taking his food, or attempting to talk, which becomes very tedious when it has to be continued for a fortnight or three weeks."

These evils, which are met with in a greater or less degree in all cases of fractnre of the lower jaw, led me to consider if some kind of instrument might not be invented, by which many, if not all of them could be guarded against. The objects I had in view were the following: "To fix the two portions of bone between two parallel forces by applying one on the teeth and the other under the base of the jaw; lastly, to keep the two portions of bone on the same vertical plane, by fixing them in a grooved plate, placed along the teeth." These Mr. Lonsdale gained by inventing the kind of instrument described and figured in his work on fractures.

"The instrument produces no inconvenience," he proceeds to say, "but gives great support to the jaw, and so much so, that some patients on whom it has been tried, have expressed a wish to have it reapplied, after it has been discontinued on account of the fracture having sufficiently united."

"The advantages gained by the above instrument appear to be the following: 1st. It is applied with much greater facility than the method of using the pieces of cork, and when once applied there is no fear of further displacement. 2d. The apparatus is applied to the lower jaw alone, which gives the patient great ease, and saves him from the necessity of keeping the mouth forcibly closed for so long a time. 3d. The motion of the lower jaw is not impeded by it, so that the patient can take his food with facility, and can talk without fear of displacing the portions of bone by which the cure is rendered much less tedious and irksome than by the ordinary method."—Lonsdale's *Treatise on Fractures*, London, 1838.

I procured one of Lonsdale's instruments and gave it a trial. While I indorse, to a great extent, much of what he claims as the advantages of the instrument, still I found it objectionable on account of its weight and size, not to say its unsightliness. I became, therefore, dissatisfied with it, and interested in seeking for some improvement upon this instrument less liable to objection, though acknowledging the inventor's merit in appreciating the true indications in the treatment and clear exposition of them. Having a case on hand at the time to treat, with the assistance of Mr. A. Wilcox, a very expert mechanical dentist, then of this city, I was enabled to perfect one to my satisfaction at least, and the contrivance represented in the above wood-cut is the result of our efforts.

This instrument, as will be seen by reference to the figure, consists, like that of Lonsdale's, of a grooved plate (A) or dental splint made of ivory, metal, or vulcanized india rubber, accurately adjusted to the teeth or alveolæ (by first taking an impression of the jaw, as is done by dentists, in wax, gutta percha, plaster of Paris, or some pliable substance), to which grooved plate are welded projecting arms (c, c), of stout iron wire, about opposite the bicuspid teeth, or a point corresponding to the corners of the mouth, arched so as comfortably to project out of the mouth without interfering with the lips. Herein the instrument differs from that of Lonsdale's, whose grooved plate is attached to a vertical bar passing over the centre of the lip, and is consequently more inconvenient to the patient than the former, the arms of which emerge *at the corners of the mouth.* The ends of the projecting arms are bent to form holes through which pieces of cord, thread, or wire are passed to attach the dental splint to a piece of wood (b), say a portion of a cigar-box cover, for a chin piece, placed under the jaw, serving the purpose of a submaxillary splint. This latter should have four holes in it, two on either side, to make it more secure.

This comprises the entire apparatus, and is applied to a fracture of the lower jaw, whenever the fracture is in the body of the bone by fitting the dental splint to the teeth, placing a compress of patent lint or some soft material between the under surface of the chin and the submental splint, then tying the tails of the thread, or twisting the wire previously passed through the holes in the latter splint, as seen in the wood-cut, to the projecting arms of the former, thus doing away with the necessity of *bandages over the head altogether.*

After trying the usual methods by bandaging unsuccessfully, as well as the instrument of Mr. Lonsdale, this apparatus was applied with the most *satisfactory and agreeable results.*

The dental splint is represented as being fenestrated; the object of this is to allow the teeth of the upper and lower jaw to dovetail, or articulate with each other, as the dentists say, in cases when there is an inequality in the size of the teeth, or irregularity in their position, so as to bring the fractured ends of the bone into perfect coaptation, without one or two projecting teeth interfering with an accurate adjustment of the fracture. If this apparatus is properly understood and applied to the fractured jaw the patient can go about his usual avocations, *take his food, and talk without fear of displacing the fragments of the broken bone*, and without the necessity of wearing a bandage or handkerchief tied over the head.

The objects had in view are precisely those laid down by Lonsdale, and the advantages, such as lightness, adaptability, simplicity and convenience, gained by this instrument over that of Lonsdale's, or any of those figured in *Hamilton on Fractures*, or in Wale's *Mechanical Therapeutics*, cannot, it appears to me, be disputed.—*Am. Jour. Med. Sciences.*

## Figures and Tables

**Figure f1:**